# Preparation of Fish Skin Gelatin-Based Nanofibers Incorporating Cinnamaldehyde by Solution Blow Spinning

**DOI:** 10.3390/ijms19020618

**Published:** 2018-02-22

**Authors:** Fei Liu, Furkan Türker Saricaoglu, Roberto J. Avena-Bustillos, David F. Bridges, Gary R. Takeoka, Vivian C. H. Wu, Bor-Sen Chiou, Delilah F. Wood, Tara H. McHugh, Fang Zhong

**Affiliations:** 1 State Key Laboratory of Food Science and Technology, Jiangnan University, Wuxi 214122, China; fliua15c@gmail.com or feiliu@jiangnan.edu.cn; 2 School of Food Science and Technology, Jiangnan University, Wuxi 214122, China; 3 Department of Food Engineering, Ondokuz Mayis University, Samsun 55139, Turkey; furkanturker61@gmail.com; 4 Western Regional Research Center, ARS, U.S. Department of Agriculture, Albany, CA 94710, USA; Roberto.Avena@ars.usda.gov (R.J.A.-B.); David.Bridges@ars.usda.gov (D.F.B.); Gary.Takeoka@ars.usda.gov (G.R.T.); Vivian.Wu@ars.usda.gov (V.C.H.W.); Bor-Sen.Chiou@ars.usda.gov (B.-S.C.); De.Wood@ars.usda.gov (D.F.W.)

**Keywords:** gelatin, nanofibers, cinnamaldehyde, solution blow spinning, antimicrobial activity

## Abstract

Cinnamaldehyde, a natural preservative that can non-specifically deactivate foodborne pathogens, was successfully incorporated into fish skin gelatin (FSG) solutions and blow spun into uniform nanofibers. The effects of cinnamaldehyde ratios (5–30%, *w*/*w* FSG) on physicochemical properties of fiber-forming emulsions (FFEs) and their nanofibers were investigated. Higher ratios resulted in higher values in particle size and viscosity of FFEs, as well as higher values in diameter of nanofibers. Loss of cinnamaldehyde was observed during solution blow spinning (SBS) process and cinnamaldehyde was mainly located on the surface of resultant nanofibers. Nanofibers all showed antibacterial activity by direct diffusion and vapor release against *Escherichia coli* O157:H7*, Salmonella typhimurium*, and *Listeria monocytogenes*. Inhibition zones increased as cinnamaldehyde ratio increased. Nanofibers showed larger inhibition effects than films prepared by casting method when *S*. *typhimurium* was exposed to the released cinnamaldehyde vapor, although films had higher remaining cinnamaldehyde than nanofibers after preparation. Lower temperature was favorable for cinnamaldehyde retention, and nanofibers added with 10% cinnamaldehyde ratio showed the highest retention over eight-weeks of storage. Results suggest that FSG nanofibers can be prepared by SBS as carriers for antimicrobials.

## 1. Introduction

Microbial contamination can not only lead to food spoilage but it can also increase the risk of foodborne illness [1]. These illnesses caused by foodborne pathogens might have several symptoms, ranging from mild gastroenteritis to life-threatening neurological, hepatic, or renal syndromes [2]. In the United States, foodborne pathogens cause around 3000 deaths each year [2,3]. There are 31 known pathogens responsible for the 9.4 million episodes of foodborne illnesses each year according to Scallan, et al. [4]. Among them, *Escherichia coli*, *Salmonella typhimurium*, and *Listeria monocytogenes* are three of the major pathogens [2,5]. One method of inactivating the foodborne pathogens and preserving food products is to add preservatives. Since consumers and food processors worry about the potential hazards of synthetic chemical residues in foods, natural additives have been gaining extensive attention [6,7].

Essential oils, containing small, hydrophobic terpenoid or phenolic compounds, have been reported to have powerful antimicrobial activity against a variety of foodborne pathogens [8,9]. Cinnamaldehyde, the main active constituent in cinnamon essential oil (60–75%), has effective activity against a broad spectrum of food-borne pathogens, including Gram-positive and Gram-negative bacteria [3,10]. It is approved by the Food and Drug Administration (FDA) as Generally Recognized as Safe (GRAS) and has been widely used in gum, ice cream, candy, beverages, breads and cereals [11]. However, the application of cinnamaldehyde in food preservation is limited by its particular flavor, volatility and lipophilic nature [1,2,12]. This limitation can be avoided or lessened by encapsulation, which can mask flavor, improve aqueous solubility and improve stability [13]. Recently, nanomaterials incorporating natural antimicrobials have attracted increased attention because they have improved antimicrobial activities as compared to traditional materials [1,14].

Nanofibers have been examined for controlled release of drugs and bioactive agents due to their large surface area to volume ratio, high encapsulation efficiency and controlled-release properties [15,16]. Among various approaches to preparing nanofibers, electrospinning has been widely used due to its relative continuous fabricating capability and simple operating process [17,18]. Previous studies have shown that cinnamaldehyde can be encapsulated into chitosan/poly (ethylene oxide) or polyvinyl alcohol nanofibers, showing excellent antimicrobial activity against *Escherichia coli*, *Pseudomonas aeruginosa,* and *Staphylococcus aureus* [1,8]. Nevertheless, the use of a large electric field, a conductive collector as well as the low fiber production rate in electrospinning limit the commercial applicability and the use for an immediate in situ application of electrospun fibers [19,20,21]. Very recently, a novel alternative technique, solution blow spinning (SBS), has been developed to produce nano- and microfibers [22,23,24,25]. Generally, a SBS setup consists of a pressurized air gas source for delivering the carrier gas and a syringe pump for polymer solution, which combines concepts of electrospinning, solution and melt spinning [21]. In addition, environmentally-friendly polymers are preferable to traditional petroleum-based polymers for use as the polymeric matrix in food applications [1]. According to Liu, et al. [26], blow spun nanofibers can be successfully prepared from fish skin gelatin (FSG) using an acetic acid solution as the solvent.

In this study, we aim to develop antimicrobial FSG-based nanofibers incorporating cinnamaldehyde at different ratios through SBS technology. Physicochemical properties of fiber-forming emulsions (FFEs) and their resultant nanofibers were investigated as a function of cinnamaldehyde ratios. Antibacterial activities of these nanofibers against *E. coli* O157:H7, *S. typhimurium*, and *L. monocytogenes* and their storage stability were evaluated. Films made by the same FFEs were investigated as controls in antimicrobial tests.

## 2. Results and Discussion

### 2.1. Characterization of Fiber-Forming Emulsions (FFEs)

#### 2.1.1. Particle Size and Particle Size Distribution

**Figure 1:**
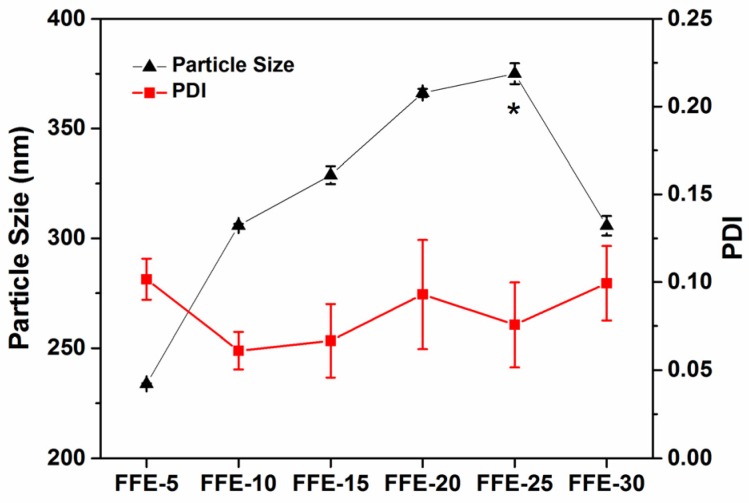
Particle size and polydispersity index (PDI) of fiber-forming emulsions (FFEs). FFE-*x*, fiber-forming emulsion prepared with the indicated cinnamaldehyde ratio to fish skin gelatin (FSG) (%). Asterisk indicates significant (*p* < 0.05) differences for particle size.

FSG and lecithin form complexes to disperse cinnamaldehyde according to Xue and Zhong [27]. As shown in Figure 1, FSG-based FFE with a particle size of 233.7 nm was obtained at the 5% cinnamaldehyde ratio. The formation of small oil droplets would be affected by oil phase composition because of its impact on oil phase and surfactant properties [28]. Therefore, the particle size was then significantly (*p* < 0.05) increased to 375.0 nm as the cinnamaldehyde ratio increased to 25%. Similar results were also reported by Liang, et al. [29] in the preparation of essential oil nanoemulsions. Further increase in cinnamaldehyde ratio to 30% led to an obvious decrease in particle size to 305.7 nm. This might be due to the presence of excessive oil volume fraction, which could not be completely encapsulated by FSG and lecithin. However, the polydispersity index (PDI) values corresponding to the particle size distribution of droplets were all lower than 0.1 at any cinnamaldehyde ratio. This suggested that all FFEs had a narrow range of size distribution. Furthermore, cinnamaldehyde might form Schiff bases with FSG due to its aldehyde structures [30], thus resulting in pink colors of FFEs especially at higher cinnamaldehyde ratios (≥15%, Figure S1).

#### 2.1.2. Viscosity

**Figure 2:**
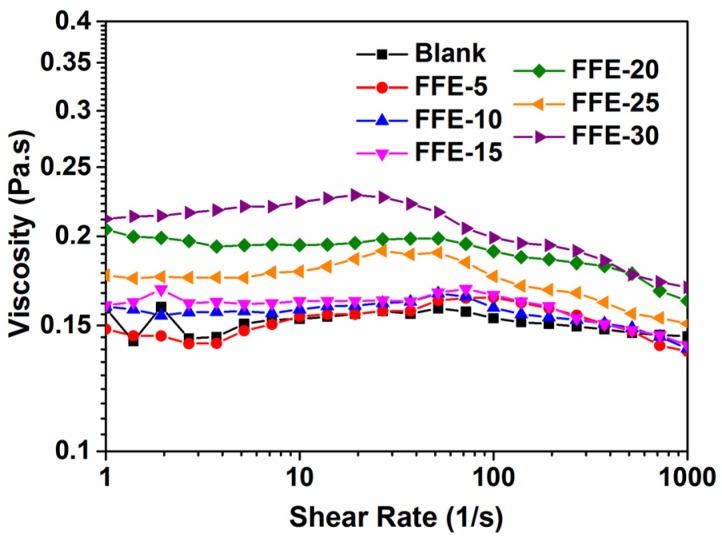
Representative viscosity of FFEs and blank as a function of shear rate (1–1000 s^−1^). FFE-*x*, fiber-forming emulsion prepared with the indicated cinnamaldehyde ratio to FSG (%). Blank is the solution without cinnamaldehyde.

The viscosity of polymer solutions had previously been shown to influence the fiber diameter, initial droplet shape and jet trajectory during the SBS process [31]. Only certain appropriate viscosities could favor the formation of fibers; decreasing the viscosity below a certain limit or increasing it above a certain limit would result in the failure of spin ability [32]. Figure 2 shows that the viscosity of FFEs slightly increased with increasing cinnamaldehyde ratio from 0% to 30%. This increase in viscosity might be due to the increase in internal cinnamaldehyde content and droplet size, as well as the expansion caused by the binding of cinnamaldehyde to gelatin [32,33]. Solutions showed shear-thinning behavior with an increase in shear rate, especially for FFE-25 and FFE-30. FFEs with higher cinnamaldehyde ratios had higher degrees of shear-thinning behavior and this shear-thinning behavior appeared at lower shear rates. This shear-thinning behavior might be due to the decrease in average cluster sizes of the droplets as the shear rate increases. In addition, the deformation or compaction of clusters could also result in the shear-thinning behavior.

#### 2.1.3. Surface Tension

**Figure 3:**
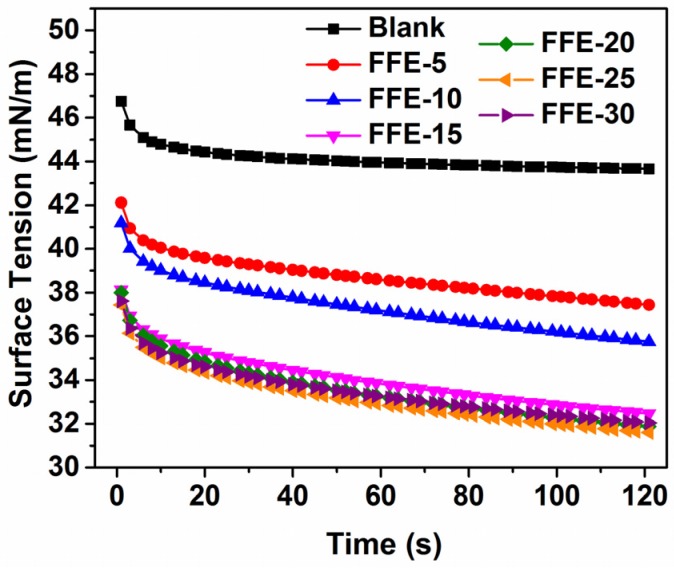
Representative surface tension of FFEs and blank as a function of time (0–120 s). FFE-*x*, fiber-forming emulsion prepared with the indicated cinnamaldehyde ratio to FSG (%). Blank is the solution without cinnamaldehyde.

The surface tension was caused by a net downward force as the liquid molecules below the surface exerted a greater attractive force than the gas molecules [34]. Similar to viscosity, it also had a direct impact on the fiber formation as well as the structures formed in the SBS process [35]. Compared to the blank sample, the surface tension of FSG solutions sharply decreased in value with the addition of cinnamaldehyde and lecithin (Figure 3). This might be due to the surface activity of lecithin, thus lowering the surface tension [32]. The increase in cinnamaldehyde ratio from 5% to 15% caused a gradual decrease in the surface tension of FFEs. This decrease in surface tension might facilitate the formation of thinner and more uniform fibers as well as reduce the formation of beads [36,37]. A relatively constant surface tension value was observed when more cinnamaldehyde (≥20%) was added to the FFEs. This might be due to the reduction in the amount of lecithin molecules available to adsorb at the air-water interface, where more FSG-lecithin complexes are needed to disperse the cinnamaldehyde at higher cinnamaldehyde ratios [27,32].

### 2.2. Characterization of Nanofibers

#### 2.2.1. Morphology and Diameter

**Figure 4:**
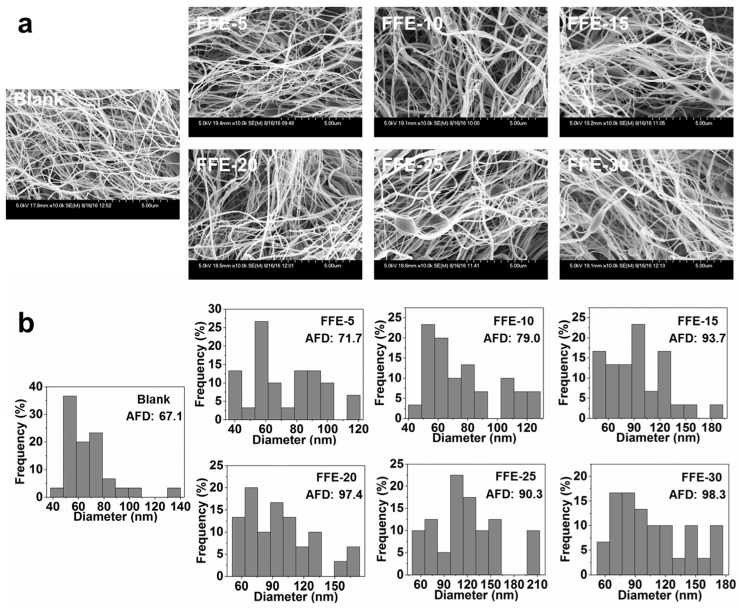
(**a**) SEM micrographs and (**b**) diameter distribution and (inset) average fiber diameter (AFD) of nanofibers prepared from FFEs and blank. FFE-*x*, nanofibers prepared with the indicated cinnamaldehyde ratio to FSG (%). Blank is the nanofibers without cinnamaldehyde. The scale bar in the bottom right of SEM image indicates 5 μm.

The morphology of FSG-based nanofibers with different cinnamaldehyde ratios (0%, 5%, 15%, 20%, 25% and 30%) was investigated using scanning electron microscopy (SEM) and the representative images and fiber diameter distributions are shown in Figure 4. Uniform nanofibers were formed at all cinnamaldehyde ratios, but beads were observed at higher ratios. The average fiber diameter (AFD) of nanofibers also increased from 67.5 nm to 98.3 nm by increasing cinnamaldehyde ratio from 0% to 30%. According to previous reports, the viscosity and surface tension of the polymer solution could affect the fiber formation in the SBS process [22,36,37]. Higher viscosity of FSG solutions could lead to less stretching of the ejected jet [16], thus forming thicker nanofibers with the presence of beads. Moreover, the increase in cinnamaldehyde ratio resulted in new morphology, with bundled nanofibers being observed. This might be due to the counteraction between viscoelasticity/surface tension from FSG solution and shearing/stretching forces from blown air [38,39]. Similar results were also reported previously [23].

#### 2.2.2. Cinnamaldehyde Distribution within Nanofibers

**Figure 5:**
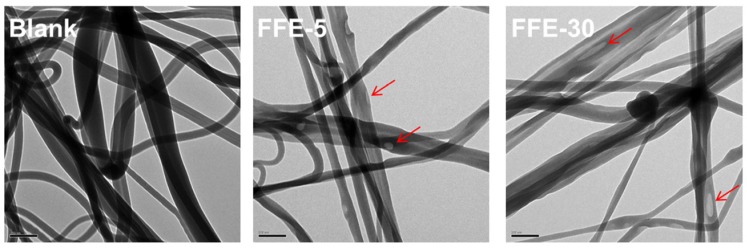
Selected TEM micrographs of nanofibers prepared from FFEs and blank. FFE-*x*, nanofibers prepared with the indicated cinnamaldehyde ratio to FSG (%). Blank is the nanofibers without cinnamaldehyde. Arrows indicated the emulsion droplets. The scale bar in bottom left indicates 200 nm.

Transmission electron microscopy (TEM) was used to visualize the distribution of cinnamaldehyde emulsions in the FSG-based nanofibers. As shown in Figure 5, the blank nanofibers showed smooth surfaces with no trace of emulsion droplets prior to the addition of cinnamaldehyde. Emulsion droplets (arrows) were exclusively observed on the surface of the FFE-5 sample in the presence of cinnamaldehyde. These droplets appeared more transparent to the electron beam in contrast to the darker nanofibers due to the differences in material contrast. It was hypothesized that the cinnamaldehyde was successfully loaded on the surface of nanofibers. Emulsion droplets became elongated as the cinnamaldehyde ratio was progressively increased to 30%. This might be due to the greater particle size (Figure 1), where emulsion droplets were sheared and stretched along the nanofibers.

**Figure 6:**
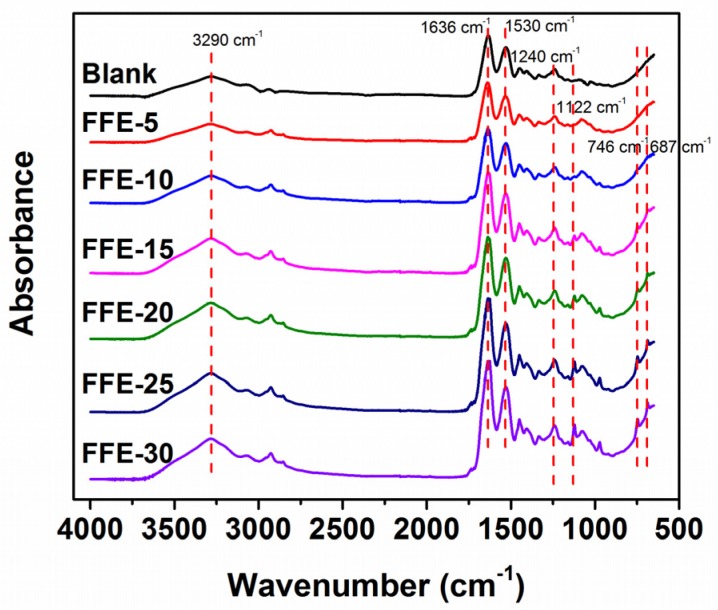
Fourier Transform Infrared Spectroscopy (FTIR) spectra of nanofibers prepared from FFEs and blank. FFE-*x*, nanofibers prepared with the indicated cinnamaldehyde ratio to FSG (%). Blank is the nanofibers without cinnamaldehyde.

Fourier Transform Infrared Spectroscopy (FTIR) was also used to confirm the presence of cinnamaldehyde in the nanofibers (Figure 6). The blank nanofibers had a broad peak at 3290 cm^−1^ corresponding to the N–H stretching coupled with hydrogen bonding. More characteristic peaks indicative of gelatin at 1636, 1530 and 1240 cm^−1^ were assigned to the C=O stretching in combination with COO, bending vibration of N–H groups associated with stretching vibrations of C–N groups, and vibrations in C–N and N–H groups of bound amide [40,41,42], respectively. These characteristic absorption peaks were not significantly modified after incorporation of cinnamaldehyde. In comparison to the spectra of blank nanofibers, cinnamaldehyde-loaded nanofibers showed some new peaks at 1122, 746 and 687 cm^−1^ corresponding to the aromatic C–H bond, –CH bending out-of-plane in aromatic ring and CH=CH bending out-of-plane in alkenes [10,43,44], respectively. These results further suggested the presence of cinnamaldehyde within nanofibers.

### 2.3. Antimicrobial Activity and Storage Stability of Nanofibers

#### 2.3.1. Antimicrobial Activity

**Figure 7:**
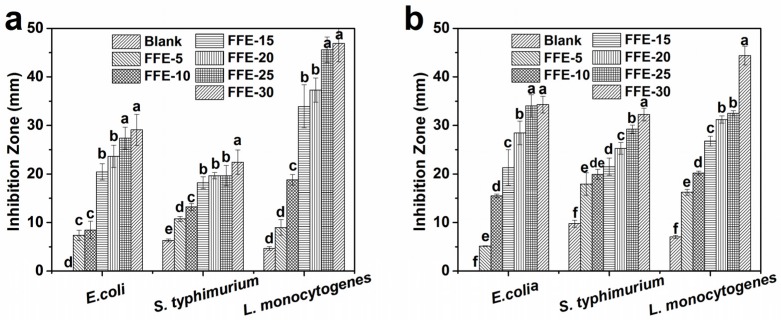
Antimicrobial activity (inhibition zone) of (**a**) nanofibers and (**b**) films prepared from FFEs and blank against *E. coli* O157:H7, *S. typhimurium*, and *L. monocytogenes*. Letters (a–f) mean significant differences (*p* < 0.05) for each foodborne pathogen. FFE-*x*, nanofibers/film prepared with the indicated cinnamaldehyde ratio to FSG (%). Blank is the nanofibers/film without cinnamaldehyde.

The antimicrobial activities of FSG-based nanofibers with and without cinnamaldehyde against *E. coli* O157:H7, *S. typhimurium*, and *L. monocytogenes* were investigated using the disk diffusion method (Figure 7a). Blank nanofibers had an inhibition effect against *S. typhimurium* and *L. monocytogenes*, which might be due to the trace amounts of residual acetic acid after fiber preparation. With the addition of 5% cinnamaldehyde, the inhibition effect was significantly enhanced (*p* < 0.05). The inhibition zone was further increased with increasing cinnamaldehyde from 5% to 30%, indicating that cinnamaldehyde was a concentration-dependent antimicrobial. Similar results were also observed by Manu, et al. [45]. In addition, *L. monocytogenes* was more sensitive to cinnamaldehyde-loaded nanofibers than *E. coli* O157:H7 and *S. typhimurium* at the same cinnamaldehyde ratios. Generally, Gram-negative bacteria have a hydrophilic outer membrane with degradative and detoxifying enzymes in periplasmic space, however, such outer membrane and protective enzymes are absent in Gram-positive bacteria [3,6,46]. This absence might have allowed cinnamaldehyde to penetrate the microbial cell easier in *L. monocytogenes* and subsequently resulted in a greater inhibitory effect.

Corresponding films with increasing cinnamaldehyde ratios were prepared from the same FFEs as controls (Figure 7b). Similar results to nanofibers were also observed in the films. Nanofibers showed smaller inhibition zones for *E. coli* and *S. typhimurium* compared to films with the same cinnamaldehyde ratios. This might be due to differences in cinnamaldehyde retention after fiber/film preparation (Table 1). More cinnamaldehyde was lost during the SBS of FFEs. However, a different behavior was observed when *S. typhimurium* was exposed to the vapor of cinnamaldehyde (Figure S2). Nanofibers showed larger inhibition zones than those of films at 25% and 30% cinnamaldehyde ratios. According to Goñi, et al. [47], an equilibrium could be attained when the cinnamaldehyde was released from nanofibers/film into the headspace in the vapor-phase test. The larger surface area to volume ratio of nanofibers might favor the sustained release of cinnamaldehyde resulting in a higher equilibrium concentration [1] and increased antimicrobial activity.

#### 2.3.2. Storage Stability

**Figure 8:**
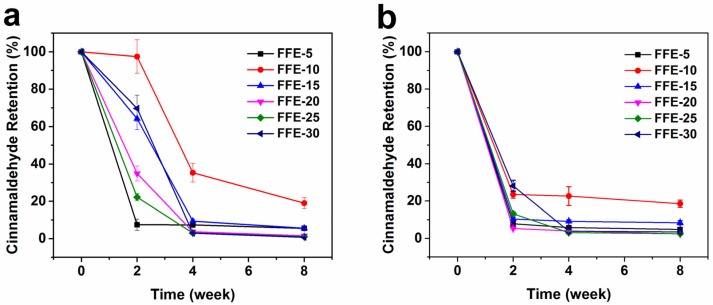
Cinnamaldehyde retention percent of nanofibers prepared from FFEs as a function of storage time. (**a**) 2 °C and 70% RH, (**b**) 20 °C and 51% RH. FFE-*x*, nanofibers prepared with the indicated cinnamaldehyde ratio to FSG (%).

The retention of volatile cinnamaldehyde in FSG-based nanofibers as a function of environmental conditions (2 °C, 70% relative humidity (RH) and 20 °C, 51% RH) over eight weeks was investigated (Figure 8 and Figure S3). At both storage conditions, nanofibers with higher cinnamaldehyde ratios showed higher remaining cinnamaldehyde contents during the first two weeks (Figure S3). After this time, the remaining contents were almost the same regardless of the cinnamaldehyde ratios. This might be due to the location of cinnamaldehyde on the surface of nanofibers, whose release was controlled by diffusion [8]. Moreover, Figure 8 showed that the cinnamaldehyde retention percent decreased as cinnamaldehyde ratio increased, however, nanofibers with 10% cinnamaldehyde ratio had the highest retention percent over eight weeks. This higher retention percent might be due to the changes in microstructure and properties of nanofibers with 10% ratio, which slowed down the cinnamaldehyde diffusion [10]. Although higher %RH might cause greater swelling and lead to a faster diffusion [48], nanofibers stored at 2 °C had better cinnamaldehyde retention than those at 20 °C for all cinnamaldehyde ratios. This suggested that temperature was the dominant factor during nanofibers storage. The higher temperature could enhance the motion of FSG chains [49], thus increasing the diffusion of cinnamaldehyde through the FSG. In real food systems, the released cinnamaldehyde could adsorb on food surfaces and exhibit antimicrobial activity during food storage.

## 3. Materials and Methods 

### 3.1. Materials

Fish skin gelatin (FSG), with high molecular weight, was provided by Norland Products Inc. (Cranbury, NJ, USA). Cinnamaldehyde, with a purity ≥98%, was obtained from Sigma Chemical Co. (St. Louis, MO, USA). Acetic acid was purchased from Avantor Performance Materials, LLC. (Baker Analyzed^®^ A.C.S. Reagent, PA, USA). Organic lecithin was supplied by Clarkson Soy Products, LLC. (Cerro Gordo, IL, USA).

### 3.2. Preparation of Fiber-Forming Emulsions (FFEs)

FFEs containing various mass ratios of cinnamaldehyde were prepared according to Table 2. Briefly, 19.8 g of FSG was dissolved in 20% (*v*/*v*) acetic acid with continuous stirring at 40 °C. Cinnamaldehyde and lecithin were then added into the FSG solution and mixed for 2 min at 15,000 rpm with a Polytron 3000 emulsifier (Kinematica, Littau, Switzerland) to form a crude emulsion according to Table 2. The crude emulsion was further homogenized three times at 15,000 psi by an M-110Y Microfluidizer (Microfluidics Corp., Newton, MA, USA) at 20 °C to form FFEs. In addition, 19.8 g of FSG was dissolved into 100.2 g of 20% (*v*/*v*) acetic acid to obtain the blank FFE. After preparation, the FFEs were stored at 4 °C for analyses or fiber formation. A camera was used to take a photo of the FFEs and blank.

### 3.3. Characterization of Fiber-Forming Emulsions (FFEs)

#### 3.3.1. Particle Size and Particle Size Distribution 

The z-average particle size and PDI of FFEs were measured via dynamic light scattering technique using a Malvern Zetasizer (Nano-ZS; Malvern Instruments, Worcestershire, UK) at 25 °C. The FFEs were diluted 100 times by ultrapure water before analysis to reduce multiple scattering effects. All measurements were performed in triplicate.

#### 3.3.2. Viscosity

The viscosity of FFEs and blank were measured with an AR 2000 rheometer (TA Instruments Inc., New Castle, DE, USA) using parallel plate geometry (diameter of 60 mm and gap of 1 mm) at 25 °C. Steady shear mode was used to measure the viscosity over a shear rate range of 1–1000 s^−1^.

#### 3.3.3. Surface Tension

The surface tension of FFEs and blank was measured with a Krüss K100 force tensiometer (Krüss GmbH, Hamburg, Germany) at 22 °C according to Parize, Oliveira, Foschini, Marconcini and Mattoso [37]. The Wilhelmy plate method was used and the surface tension was recorded for 120 s after placing the platinum plate on the solution surface.

### 3.4. Preparation of Nanofibers

Nanofibers were prepared with the solution blow spinning (SBS) method at 18 °C and 48% relative humidity (RH) according to our previous study [26], with minor modifications. The SBS apparatus consisted of a syringe pump (KD Scientific, Holliston, MA, USA), a concentric nozzle, and a source of compressed air. FFEs and blank were fed through a 10 mL Becton-Dickinson (Franklin Lakes, NJ, USA) plastic syringe to the inner nozzle. The feed rate was controlled at 0.1 mL/min by the syringe pump. Pressurized air was delivered through the outer nozzle with a pressure of 0.38 MPa and an airflow speed of 14 m/s. A static plate collector covered with aluminum foil located 50 cm from the nozzle tip was used to collect nanofibers. Nanofibers were sealed in Petri dishes, covered with aluminum foil and stored at 4 °C until testing.

### 3.5. Characterization of Nanofibers

#### 3.5.1. Scanning Electron Microscopy (SEM)

Nanofibers were cut and glued onto aluminum specimen stubs using double-sided adhesive carbon tabs. The samples were first coated with gold-palladium on an Emitech K550X sputter coater (Quorum Technologies Inc., Guelph, ON, Canada) at 25 mA for 45 s. They were then examined using a Hitachi S-4700 field emission scanning electron microscope (Hitachi, Tokyo, Japan) at an accelerating voltage of 5 kV. Nanofiber diameters were measured directly from the scanning electron micrographs using Image Pro Plus 7 (Media Cybernetics, Inc., Rockville, MD, USA) software. The average fiber diameter (AFD) was calculated from ~100 random measurements.

#### 3.5.2. Transmission Electron Microscopy (TEM)

Nanofibers were first applied to a 200 mesh copper grid coated with Formvar/carbon (Catalog No. 01801, Ted Pella Inc., Redding, CA, USA). TEM was then analyzed using a Hitachi H-700 transmission electron microscope (Hitachi, Tokyo, Japan) with an accelerating voltage of 100 kV at room temperature. The samples were not stained.

#### 3.5.3. Fourier Transform Infrared Spectroscopy (FTIR)

FTIR spectra of nanofibers were investigated using an FTIR spectrometer (Nicolet IS 10, Thermo Fisher Scientific Inc., Madison, WI, USA) with attenuated total reflection (ATR) mode at room temperature. Each spectrum was recorded from 700 to 4000 cm^−1^ using 64 consecutive scans with a resolution of 4 cm^−1^. Spectra were then analyzed using OMNIC 8.2 data collection software (Thermo Fisher Scientific Inc., Madison, WI, USA).

### 3.6. Antimicrobial Activity and Storage Stability of Nanofibers

#### 3.6.1. Antimicrobial Activity

Frozen cultures of *E. coli* O157:H7 ATCC^®^ 35150, *S. typhimurium* ser. ATCC^®^ 14028 and *L. monocytogenes* ATCC^®^ 19115 from the Western Regional Research Center (USDA;ARS:WRRC) strain collection were streaked on tryptic soy agar (TSA). TSA was composed of tryptic soy broth and 1.5% granulated agar (Difco, Becton, Dickinson & Co., Sparks, MD, USA). TSA with pathogens were then incubated at 37 °C for 24 h. After that, TSA was used to re-streak an isolated colony and incubated at 37 °C for 24 h. A tube with 5 mL Trypticase Soy Broth (TSB, Difco) was also used to inoculate an isolated colony, which was incubated at 37 °C for 24 h with agitation. This microbial broth was then serially diluted (10-fold) using 0.1% peptone water.

Each bacterial culture with a 0.1 mL volume of 1 × 10^5^ colony-forming-units (CFU)/mL was added onto each of five TSA plates. Each plate was spread evenly with the inoculum and was then dried in a biosafety hood for 5 min. For diffusion release, each agar plate was divided evenly into 2–4 areas and labeled with the different antimicrobial concentrations. On the center of each area, ~15 mg nanofiber mat was deposited in a constant area over the inoculated agar allowing all fibers to touch the agar by applying slight pressure to avoid breaking the agar gel. The plates were incubated at 35 °C for 24 h. The inhibition diameter (colony-free perimeter), including the area where the fibers were deposited on the agar surface, was measured with a digital caliper in triplicate after 24 h of incubation and reported in mm.

#### 3.6.2. Storage Stability

The loss of cinnamaldehyde from nanofibers during eight-weeks of storage at 2 °C (70% RH) and 20 °C (51% RH) under constant air flow was measured at two week intervals. Since volatile cinnamaldehyde would occupy the surrounding headspace of vials to inhibit release, the nanofiber samples were stored without covering. Cinnamaldehyde retention in the FSG-based nanofiber matrix was measured by gas chromatography. Around 20 mg of nanofibers were extracted with 10 mL of dichloromethane containing 0.1013 mg/mL of cinnamyl alcohol (internal standard). The % recovery of the first extraction was 93.1% (average of two replicates) as determined by the analysis of three consecutive extractions. Extracts containing cinnamaldehyde were analyzed with an Agilent 6890 GC equipped with a flame ionization detector (FID). A 30 m × 0.32 mm i.d. (df = 0.25 µm) DB-5 bonded-phase fused-silica capillary column (Agilent Technologies Inc., Folsom, CA, USA) was employed. The injector and detector temperatures were 220 °C. The oven temperature was programmed from 80 °C to 165 °C at 6 °C/min. The linear velocity of the helium carrier gas was 38.2 cm/s (80 °C). Split injections were used with a split ratio of 1:7.9. The instrument was controlled and data were processed by an HP ChemStation (Rev. B.01.01[164]SR1; Agilent Technologies Inc., Santa Clara, CA, USA).

The relative response factor (RRF) was calculated with the following Equation (1):

*RRF* = (*Aa*/*Ais*) × (*Cis*/*Ca*) (1)

where:

*Aa* is the GC peak area of analyte

*Ais* is the GC peak area of internal standard

*Cis* is the concentration of internal standard in a solution

*Ca* is the concentration of analyte in a solution.

The relative response factor, *RRF*, of cinnamaldehyde/cinnamyl alcohol was determined to be 1.27.

The concentration of cinnamaldehyde was calculated with the following Equation (2):

*Ca* = ((*Aa*/*Ais*) × (*Cis*/*RRF*))/% recovery (2)

### 3.7. Statistical Analysis

Data were reported as mean value ± standard deviation. One-way analysis of variance (ANOVA) was used to analyze the data with the SPSS 19.0 package (IBM, New York, NY, USA). The significant differences of the mean values (*p* < 0.05) was determined by Duncan’s-multiple range test.

## 4. Conclusions

FSG-based nanofibers incorporating cinnamaldehyde were successfully prepared via SBS. A significant influence of cinnamaldehyde ratio was demonstrated from the physicochemical, rheological and antimicrobial properties of FFEs and their nanofibers. The morphology of nanofibers was dependent on the viscosity of FFEs, which was controlled by the cinnamaldehyde ratio. Cinnamaldehyde droplets were stretched and distributed on the surface of nanofibers due to their larger size compared to prepared nanofibers. SBS resulted in higher losses of cinnamaldehyde from FFEs than those during film casting. All nanofibers and films showed antimicrobial activity by diffusion and vapor release against three selected foodborne pathogens at all concentrations of cinnamaldehyde. Since cinnamaldehyde was a concentration-dependent antimicrobial, more cinnamaldehyde led to enhanced inhibition effects. Nanofibers had higher antimicrobial activity than films in vapor phase antimicrobial tests due to their larger surface area to volume ratios. The diffusivity of cinnamaldehyde from nanofibers was greatly affected by temperature during storage tests. Loss of cinnamaldehyde was higher at higher cinnamaldehyde ratio, but nanofibers with 10% cinnamaldehyde ratio had the highest retention percent due to changes in their microstructure properties. This research provides insights on the factors that influence the formation and antimicrobial efficacy of cinnamaldehyde-loaded blow spun nanofibers for controlled-release applications.

## Figures and Tables

**Table 1:** Cinnamaldehyde retention of freshly prepared fibers or films. FFE-*x*, nanofibers prepared with the indicated cinnamaldehyde ratio to FSG (%). Blank is the nanofibers without cinnamaldehyde.

Samples	Cinnamaldehyde Retention after Fiber Preparation (mg C/mg F)	Cinnamaldehyde Retention after Film Preparation (mg C/mg F)
Blank	-	-
FFE-5	0.013	0.002
FFE-10	0.004	0.044
FFE-15	0.012	0.021
FFE-20	0.058	0.074
FFE-25	0.080	0.116
FFE-30	0.084	0.171

**Table 2:** Formulation of FFEs and blank. FFE-*x*, fiber-forming emulsion prepared at the indicated cinnamaldehyde ratio to FSG (%). Blank is the solution without cinnamaldehyde.

Ingredient (g)	Blank	FFE-5	FFE-10	FFE-15	FFE-20	FFE-25	FFE-30
FSG	19.8	19.8	19.8	19.8	19.8	19.8	19.8
20% acetic acid	100.2	96.24	95.25	94.26	93.27	92.28	91.29
Cinnamaldehyde	-	0.99	1.98	2.97	3.96	4.95	5.94
Lecithin	-	2.97	2.97	2.97	2.97	2.97	2.97
Total	120	120	120	120	120	120	120

## Supplementary Materials

Supplementary materials can be found at http://www.mdpi.com/1422-0067/19/2/618/s1.

## Abbreviations

FSG	fish skin gelatin
FFE	fiber-forming emulsion
SBS	solution blow spinning
FDA	Food and Drug Administration
GRAS	Generally Recognized as Safe
PDI	polydispersity index
RH	relative humidity
SEM	scanning electron microscopy
AFD	average fiber diameter
TEM	transmission electron microscopy
FTIR	fourier transform infrared spectroscopy
CFU	colony-forming-units
FID	flame ionization detector
RRF	relative response factor
ANOVA	analysis of variance
