# Supplementary material for: Preparation of Fish Skin Gelatin-Based Nanofibers Incorporating Cinnamaldehyde by Solution Blow Spinning
Source: Int J Mol Sci. 2018 Feb 22;19(2):618. doi: 10.3390/ijms19020618 (PMC5855840; doi:10.3390/ijms19020618)
Supplement: Supplementary file 1 [file ijms-19-00618-s001.pdf]

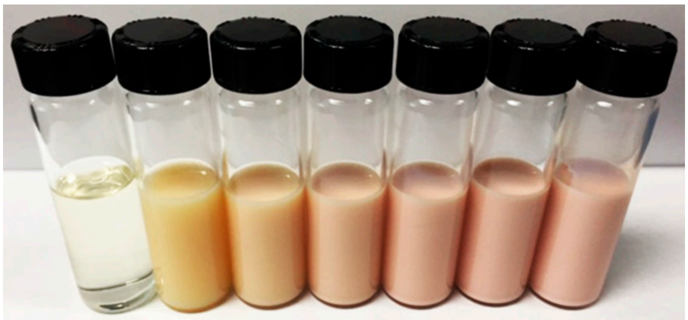

Figure S1. Appearance of FFEs and blank solutions. From the left to the right is the blank, FFE-5, FFE-10, FFE-15, FFE-20, FFE-25 and FFE-30, respectively. FFE-x, fiber-forming emulsion prepared with the indicated cinnamaldehyde ratio to FSG (%). Blank is the solution without cinnamaldehyde.

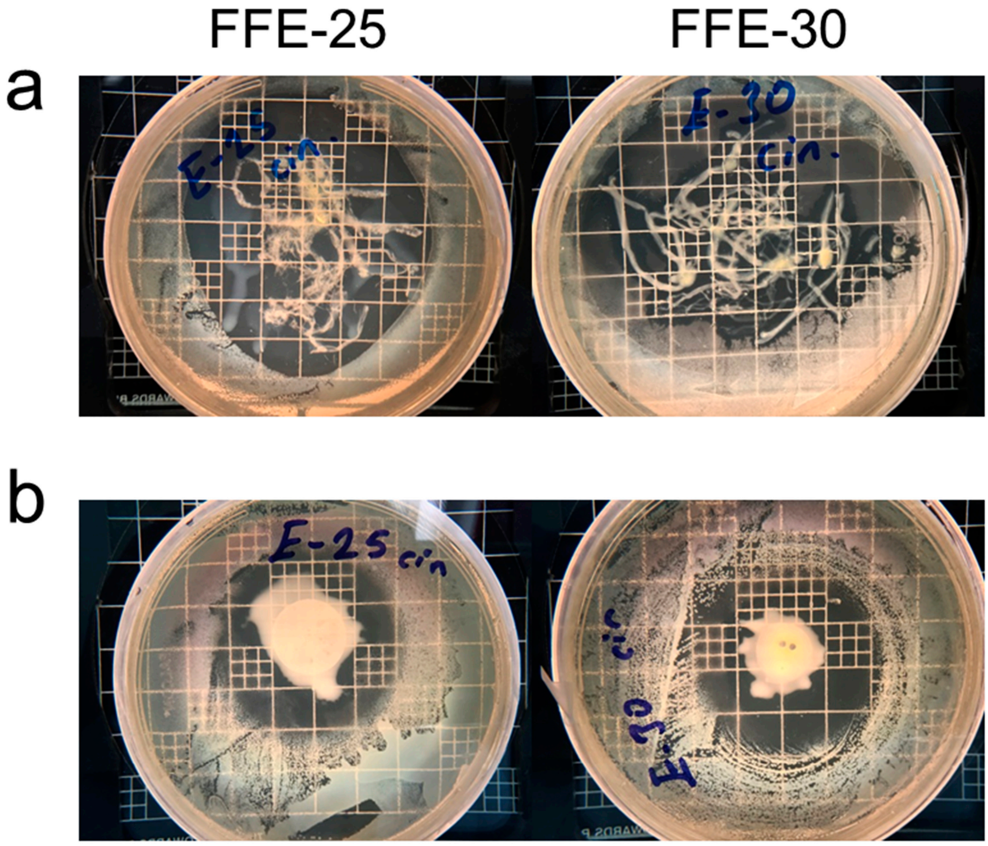

Figure S2. Vapor phase antimicrobial activity of (a) nanofibers and (b) films prepared from FFEs and blank against *S. typhimurium*. FFE-*x*, nanofibers/films prepared with the indicated cinnamaldehyde ratio to FSG (%). Blank is the nanofibers/films without cinnamaldehyde.

For vapor phase analysis, Petri dish lids were covered with ~30 mg fibers, and fibers were slightly pressed with a spatula in order to stick them to lids. 14 mL of sterilized TSA were poured into dishes and dried in hood without closing the lids. After drying 0.1 mL of second dilution of S. enterica culture were spread on to agar and closed with lids down. All dishes were covered with Parafilm in order to inhibit vapors leaking out. Each fiber was analyzed in duplicate for each strain.

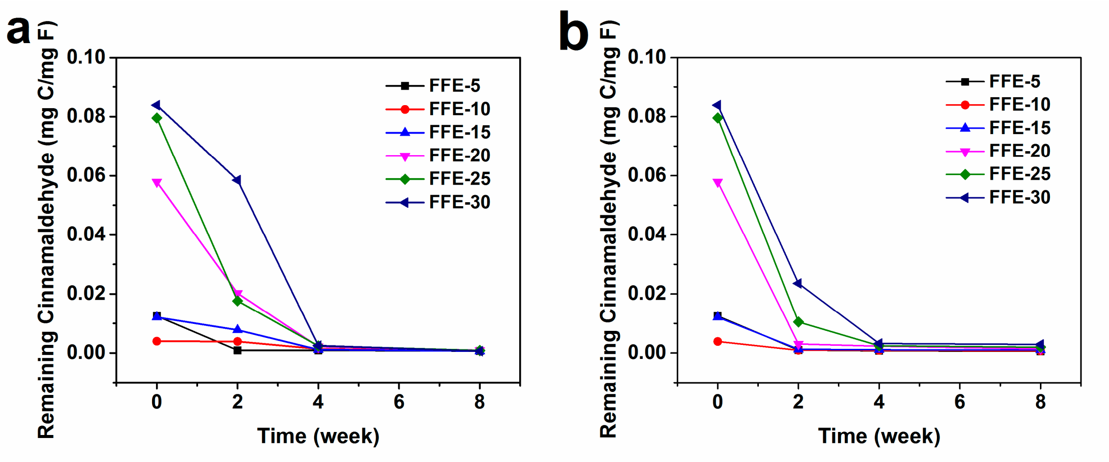

Figure S3 Cinnamaldehyde remaining in nanofibers prepared from FFEs as a function of storage time. (a) 2 °C and 70% RH, (b) 20 °C and 51% RH. FFE-*x*, nanofibers prepared with the indicated cinnamaldehyde ratio to FSG (%).
